# Brachymetatarsia: Surgical Management, Case Report, and Literature Review

**DOI:** 10.1155/2022/8253096

**Published:** 2022-03-10

**Authors:** David Zhu, Maxime Lefèvre, Andréa Fernandez, Laurent Galois

**Affiliations:** Centre Chirurgical Emile Gallé, CHU Nancy, 49 Rue Hermite, 54000 Nancy, France

## Abstract

**Background:**

Brachymetatarsia is defined by an abnormal shortening of the metatarsal bone. This rare condition is mostly primary and congenital. Consequences of this malformation are both esthetic and functional, due to pain and mechanical problems in the forefoot. Surgical management is an important part of patient care. There are two main options: gradual lengthening by progressive callotosis distraction using an external fixator and one stage lengthening using bone graft and osteotomy of the bone. This review presents two cases using the one stage lengthening surgical management method. We also discuss some reports in the literature with the aim to compare the advantages and disadvantages of the two surgical methods. Literature concerning the surgical management of brachymetatarsia was identified using the PubMed and Google Scholar databases. *Patient Presentation*. We describe two female patients aged 20 and 26 years who underwent one stage lengthening surgery of the fourth toe with isolated brachymetatarsia using an iliac bone graft and internal fixator plate. The two patients had a lengthening of around 10 mm after postoperative evaluation. No skin complications were noted, but one of the patients reported flexor stiffness after surgery. Concerning the functional and cosmetic aspects, the two patients are satisfied with the management.

**Conclusions:**

In the literature, one stage lengthening seems to be the most favorable option for the care of brachymetatarsia. Studies show a short healing time and fewer complications like infection, stiffness, malalignment, and malunion. Some reviews note the utility of the gradual lengthening of severe brachymetatarsia when a longer lengthening is necessary. There is no definite consensus concerning the management of brachymetatarsia.

## 1. Introduction

Brachymetatarsia is a rare malformation characterized by an abnormal shortening of one or more toes. The condition is defined by the presence of shortness of more than 5 mm of the metatarsal arch [1, 2]. The incidence reported in the literature is between 0.02% and 0.05%, and the condition is more frequent in females, with a ratio of 25 : 1 [2–5]. Brachymetatarsia appears to be the consequence of retarded growth or premature closure of the epiphyseal plate [6]. The etiology can be congenital and idiopathic, posttraumatic, postinfection, iatrogenic, or secondary to a systemic disease such as malignancy, sickle cell disease, pseudohyperparathyroidism, Turner's syndrome, Down's syndrome, Apert syndrome, athyroidism, or osteodystrophy.

The majority of cases affect the fourth metatarsal and between 36% and 72% of cases are bilateral [1, 6, 7]. The clinical presentation is most commonly cosmetic complaint secondary to the deformity. Moreover, some patients report pain caused by the mechanical dysfunction on the forefoot [8, 9].

Treatment of brachymetatarsia is conservative or surgical. Conservative treatment consists of wearing accommodative shoes; however, this does not resolve the cosmetic issue. Different surgical treatments are described: the gradual lengthening distraction by callotosis and one stage lengthening. However, there is currently no consensus on the surgical management as each treatment has its advantages and disadvantages.

The aim of this study is to describe the global outcomes of the surgical management of brachymetatarsia according to the literature and to present, based on our experience, two cases using one stage lengthening treatment of this rare malformation.

## 2. Cases

The research findings related to brachymetatarsia were identified using the PubMed and Google Scholar databases, using “congenital” and “brachymetatarsia” as keywords. Published data in any language describing surgical techniques of gradual lengthening or single-stage lengthening were selected.

We describe two female patients aged 20 and 26 years, who underwent one stage lengthening surgery with an iliac bone graft. We retrospectively reviewed the two cases with a one-year follow-up. Patients provided written informed consent for the case details to be published. All data have been anonymized. Patient 1 had an isolated shortening of 10 mm of the fourth toe on the right foot. The main complaint was esthetic, with an important mental effect. Patient 2 had bilateral brachymetatarsia of the fourth toes. The right foot presented only an esthetic issue, but there was functional dysfunction associated with pain in the left. Only the left foot has been operated on to date. The left fourth toe presented a 15 mm defect.

**Figure 1:**
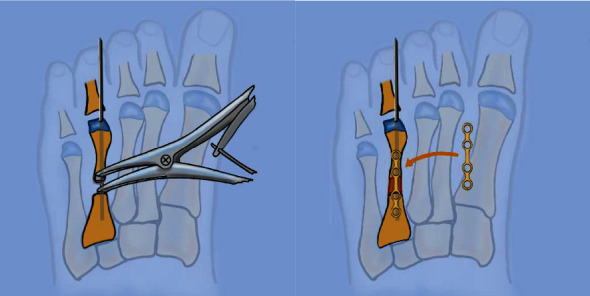
Illustration showing correction of fourth toe brachymetatarsia. (a) Distraction of the bone with cloward after shaft osteotomy after insertion of a 1.2 mm K-wire. (b) Iliac bone graft placed in the osteotomy and fixation with a LCP foot plate locked by screws.

The intraoperative procedure was performed under general anesthesia in the supine position. A pneumatic tourniquet was applied to the calf. The homolateral iliac crest was exposed for the graft. A Z-plasty skin incision was made on the dorsal surface of the fourth metatarsal. After exposition of the extensor digitorum longus tendon, a tenotomy was performed to permit the soft tissue lengthening. The flexor tendon was preserved. Osteotomy was performed on the medium shaft of the fourth metatarsal with an oscillating saw (Figure 1). The distraction was first performed using a cloward distractor and aided by inserting a 1.2 mm K-wire in the longitudinal axis. An autogenous iliac bone graft was taken from the homolateral iliac crest on the internal table to preserve the contour of on the external iliac chest. The graft was modeled according to the preoperative distraction. A specific locking foot plate was used to fix the distraction, and locked screws were positioned at the proximal and distal part of the plate. The graft was placed under the plate between the proximal and distal fragments of the osteotomy. Fluoroscopic control was used to monitor the per-operative lengthening. The pneumatic tourniquet was released to check the distal toe circulation and coloration. The Z-plasty was then closed. A compressive dressing and a short posterior leg splint were placed directly after surgery. The splint was immobilized by a circular resin boot. Weight bearing on the operated feet was forbidden for one month.

## 3. Results

**Figure 2:**
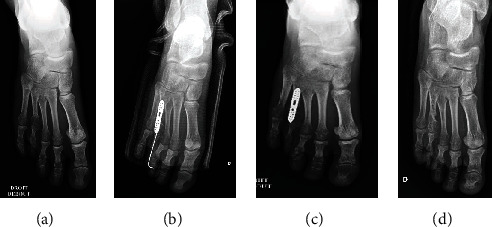
Case 1, a 20 years old female with a brachymetatarsia of the right fourth toe. (a) Preoperative X-ray of the right foot showing the shortness of around 10 mm. (b) Postoperative X-ray of the right foot. (c) Postoperative X-ray after K-wire removal at 1-month follow-up. (d) Postoperative X-ray after plate removal at 1-year follow-up.

**Figure 3:**
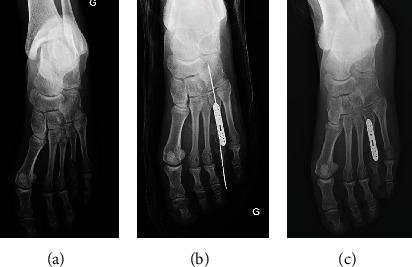
Case 2, a 26-year-old female with bilateral brachymetatarsia of the fourth toes who underwent surgical management of the left foot. (a) Preoperative X-ray of the left foot showing a shortness of the fourth toe of around 10 mm. (b) Postoperative X-ray of the left foot. (c) Post-operative X-ray after the K-wire removal at 1-month follow-up.

**Figure 4:**
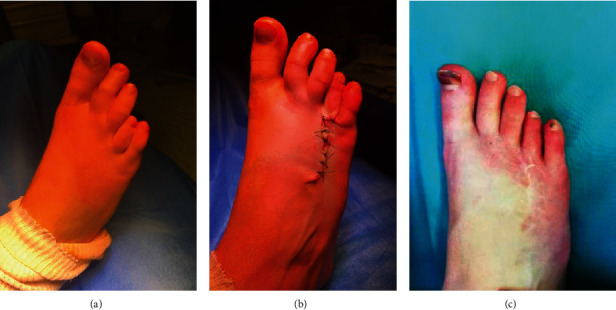
Case 1. (a) Preoperative photography. (b) Postoperative photography after closing the Z-plasty. (c) Photography of the lengthening at 12-month follow-up.

Patients were evaluated using functional, esthetic, and radiologic assessment at 1 month, 3 months, and 6 months. No skin healing complications were reported. K-wires were removed at 1 month after radiologic assessment. Progressive weight bearing was started at the 1 month assessment. The two cases (Figures 2–4) maintained a lengthening of 10 mm during the follow-up period. Both operated toes were aligned and harmonious with the other toes. No pain was reported during the follow-up. However, at 3 months, patient 1 reported a stiffness of the fourth toe with a plantar overbalance caused by the lengthening of the extensor tendon. Additionally, the LCP plate caused discomfort on wearing shoes, but no pain. The removal of the material associated with a flexor tenoplasty was planned at 1 year. Patient 2 did not report any complications; the lengthening of the right toe that presents a brachymetatarsia is planned.

## 4. Discussion

Brachymetatarsia is a rare condition. The data collected from the literature describe the surgical techniques, complications, advantages, and disadvantages regarding the correction of the malformation. Surgical treatment is commonly performed in patients older than 12–14 years, because the malformation is not obvious before this age [10, 11]. Most of the surgeries were performed at the request of the patient due to cosmetic concerns. The mechanical pain caused by the malformation can be treated using conservative treatment.

The two most widely used techniques for treatment are gradual lengthening using an external fixator and single stage lengthening. The gradual lengthening method is well documented in the literature and is used by most surgeons [8, 10].

Our patients underwent one stage lengthening using a bone graft and plate fixation after osteotomy. The surgery provides desirable cosmetic and functional results, with a lengthening of around 10 mm in both cases. The healing time was short, with weight bearing authorized after 1 month. Despite the fact that case 1 presented a stiffness of the fourth toe, the functional aspect was preserved. Additionally, no pain was noticed after surgery, and the two cases were satisfied with the global outcome, with one of the cases even waiting for the contralateral surgery.

Some studies have reported the advantages of one stage lengthening [1, 11–13]. The advantages of this technique are principally a short treatment period and easier post-operative recovery. Additionally, the use of a plate and k-wire for the osteosynthesis offer an immediately stable bone graft. Some cases reported in the literature did not use a plate and used only k-wire [4, 12, 14]; in these cases, no complications such as secondary shifting were reported, but these studies concern only small series. Concerning the bone graft, the donor site should be considered; studies reported metatarsal, calcaneal, fibula, and iliac crest bone grafts, and no study compared the benefits of different sites [4, 12–15].

Gradual lengthening using an external fixator was the first surgical management for brachymetatarsia to be developed. The surgery is performed after the osteotomy, to stabilize the distraction with an external fixator using pins at the proximal and distal fragment. The external fixator is progressively distracted over a number of weeks and is removed when the lengthening is satisfactory and after radiographic assessment showing bone consolidation. The major advantage of callus distraction over one stage bone grafting is that the lengthening of the metatarsal can be increased. In the review of the literature, the mean lengthening of the callus distraction is 18.55 mm (range 15 to 20 mm) and for the one stage procedure is 12.6 mm (range 9.0 to 20.9 mm) [1, 2, 4, 5, 8, 10–25]. Additionally, the progressive distraction has a second advantage: the simultaneous lengthening of the bone and the soft tissue can avoid the stress caused to the toe. Indeed, the major complication of one stage lengthening is the stiffness of the metatarsophalangeal joint after the bone graft [4, 12, 15]. Callotosis distraction is associated with a longer healing time because of the progressive lengthening and the removal of the material after the bone consolidation. In addition, the presence of the material can be an esthetic and emotional problem during the healing time. Also, the distraction needs frequent follow-up, more than single stage lengthening.

The most frequently observed complications after the callus are metatarsal malalignment, malunion, angulation, subluxation and dislocation, failure of metatarsal bone formation, fracture, infection, arthrosis, stiffness of the MTP joint, and skin breakdown (1,5,6,24). To prevent metatarsal displacement, some authors recommended temporary K-wire fixation during the distraction period [6].

Concerning the one stage procedure, the complications are stiffness, pseudoarthrosis, nonunion of the osteotomy, and pain. A few cases of metatarsal postoperative malformation are reported in the literature, but they are rare [1, 12, 26]. There were no donor site complications found in the literature. Some reviews mention neurovascular complications after bone grafting, but we did not find this type of complication in all case reports.

A limitation of these studies is that only a few assess a large number of patients, and most of the publications are case reports. We did not find level 1 or 2 studies concerning brachymetatarsia management. Because of the rarity of the deformation, a publication bias is present due to the fact that only reports with good results are published. Jones et al. [1] published a retrospective review of 457 brachymetatarsia cases, and Lee et al. [26] published a retrospective review of 153 toes, comparing the outcomes of the single stage versus callus distraction using external fixator methods. Despite the large number of patients, both studies showed similar results to case report series.

Regarding the functional aspect, some studies used AOFAS (American orthopedic foot and ankle score) scores to evaluate preoperative and postoperative feelings of patients, showing an improvement of the score after operation [2, 4, 5, 12, 26]. These results show the utility of the surgery and surgical management. There is no consensus to date. Some authors recommended a maximum lengthening of 15 mm for the bone graft [5, 12, 26, 27], with the surgery personalized according to the requirement for lengthening. However, this review, like others, demonstrates the greater complication rate, the longer healing time of the callus distraction, and the advantage of the single stage bone graft when a lesser degree of lengthening is necessary. Currently, the choice of technique is up to the surgeon and the patient.

Large prospective studies with selective inclusion criteria and statistical analysis are needed to compare the outcomes and complications of the technique during a long follow-up.

## 5. Conclusion

In the literature, one stage lengthening seems to be the most favorable option for the treatment of brachymetatarsia. Studies show a short healing time and fewer complications like infection, stiffness, malalignment, and malunion. Some reviews note the utility of the gradual lengthening of severe brachymetatarsia when a greater degree lengthening is necessary. There is no consensus concerning the management of brachymetatarsia.
